# Metal Scrap to Hydrogen: Manufacture of Hydroreactive Solid Shapes via Combination of Ball Milling, Cold Pressing, and Spark Plasma Sintering

**DOI:** 10.3390/nano13243118

**Published:** 2023-12-11

**Authors:** Olesya A. Buryakovskaya, Mikhail S. Vlaskin, Aleksey V. Butyrin

**Affiliations:** Joint Institute for High Temperatures of the Russian Academy of Sciences, 125412 Moscow, Russia; aleksey.butyrin@yandex.ru

**Keywords:** aluminum–copper composite, ball milling, cold pressing, spark plasma sintering, hydrogen generation, waste utilization

## Abstract

Two sorts of tablets were manufactured from ball-milled powder (aluminum scrap and copper) by cold pressing and spark plasma sintering. Their microstructure, phase, and elemental compositions were investigated via scanning electron microscopy, X-ray diffraction analysis, and energy-dispersive X-ray spectroscopy. New phases, Al_2_Cu and MgCuAl_2_, were detected in the samples. Their microstructure was formed by welded scrap particles, the intermetallides, and Cu-rich regions located majorly along ‘interparticle boundaries’ and, to a lesser extent, within small, micro- and nanosized ‘intraparticle spots’. The tablets were sealed with adhesive, so only the top surface was exposed to the environment, and tested in a chlorine aqueous solution for hydrogen generation performance. For both sample sorts, hydrogen yields of nearly 100% were achieved. The sintered tablets reacted faster than the cold-pressed ones: at 60, 70, and 80 °C, their entire ‘conversion into hydrogen’ took ~80, 40, and 30 min. vs. ~220, 100, and 70 min. The experimental kinetic curves were fitted with a contracting geometry equation, and those for the sintered samples were approximated with higher precision. The key effect of the additive was to enhance hydrogen evolution through the galvanic corrosion of Al in the regions adjacent to the intermetallic inclusions and Cu-rich spots.

## 1. Introduction

In recent decades, a great number of countries have faced economic, social, and ecological problems associated with the power sector’s dependence on fossil fuels [1,2]. The said problems became drivers for the global transition from the use of carbon-based combustibles towards a sustainable power supply based on renewable energy sources and clean, ‘green’, energy carriers, with hydrogen considered a key alternative. Although the current large-scale hydrogen production predominantly relies on fossils both as raw materials and energy sources, a wide variety of renewables (e.g., wind, solar, and geothermal power) have been tested as promising power sources for its generation by water splitting [3].

However, the existing problems relating to the safe and effective storage and transportation of this explosive gas that leaks easily into the atmosphere still remain unsolved. Major efforts have been made to design high-pressure vessels with working pressures of 700 MPa or higher [4], cryogenic tanks operating at 20 K [5], and metal hydrides [6]. However, the respective downsides of the listed methods are potential degradation and/or damage of the polymer liner (type IV hydrogen storage tanks) [7], considerable energy consumption and mass loss of liquid hydrogen [8], and either relatively low overall hydrogen gravimetric capacity (below 2 wt.%) or reversibility issues [9].

Hydrogen, in a desired amount, can be obtained in situ as well. Some metals (e.g., Mg and Al) can potentially react with water with hydrogen evolution quite readily but not uncontrollably, as in the case of alkali metals. However, common practice shows that under standard conditions, those metals either are protected by a dense oxide layer hampering water penetration or promptly get coated with a poorly permeable layer of reaction products [10,11,12,13]. To overcome the said limitations, a good deal of activation methods were developed aimed at dissolving, removing, destructing, or disrupting the said passivation layers or enhancing water diffusion through them. Water diffusivity is known to increase with an increase in reaction temperature. Thus, for dispersed aluminum (micropowders), high hydrogen yields were obtained from their reactions with saturated steam and water within the pressure and temperature ranges of 12–15 MPa and 300–340 °C, respectively [14,15]. Bulk samples (granules, various waste pieces, etc.) were also successfully transformed into hydrogen and reaction products under temperatures ranging from 280 to 400 °C [16,17]. In the study [18], a significant effect of aluminum purity on its conversion into hydrogen under hydrothermal conditions (200–280 °C) was established. Due to their small sizes, aluminum nanoparticles were thoroughly oxidized by water at room temperature [12,19]. The mentioned methods are suitable for obtaining pure hydrogen and reaction products. Another widely known approach is the implementation of acidic and alkali solutions (HCl, NaOH, KOH, etc.) for ‘chemical’ activation, which is especially beneficial for coarse samples and waste multilayer packaging materials [20,21,22,23,24,25]. The elaboration of composite aluminum and magnesium-based materials by ball milling with various additives encompasses a big cluster of techniques for the effective removal, disruption, and destruction of the passive layers. They include high-energy ball milling of the hydroreactive metals without additives [26,27], with various salts (NaCl, KCl, AlCl_3_, MgCl_2_, etc.) [28,29,30], carbon-based materials, metal oxides and hydroxides [31,32,33,34,35], metals (Bi, Zn, Sn, In, Pb, Ga, Ca, Ni, etc.), and alloys [18,27,36,37,38,39,40]. Ball milling results in the formation of lattice structure imperfections that are vulnerable to corrosion attacks. The addition of brittle substances (salts and oxides) promotes particle size reduction during ball milling, thus increasing the specific surface area of a sample. In conductive media, the oxidation of Al and Mg ‘mechanically coupled’ with ‘nobler’ metals is accelerated by galvanic corrosion. The latest activation methods were successfully used for the effective utilization of aluminum- and magnesium-based waste materials with hydrogen generation [41,42,43,44].

A number of models have been proposed for the simulation of the metal oxidation process with hydrogen generation. In the study [16], a first-order reaction model was used to approximate kinetic curves for aluminum powder, casing, and foil reacting with water under hydrothermal conditions. For commercial Al powders (‘ASD-1’, ‘ASD-4’, and ‘PAP-2’ grades) reacting with water at 100 °C, a ‘shrinking sphere’ equation was found to be applicable to the kinetics description at the initial stage, and the second stage was approximated using the parabolic diffusion equation [45]. For Al powder reacting with Na_2_SnO_3_ (0.05 M), a similar ‘shrinking sphere’ model was used at the first stage, and the Ginstling–Brounshtein equation was employed for the second one [46]. A ‘shrinking core’ model was applied to the Al–H_2_O reaction in cases of spherical Al powder particles [47,48]. It was further improved and successfully applied to the case of ball-milled Al particles [49]. For Al foil reacting with a low alkaline NaOH aqueous solution, its oxidation process was fitted with the linear–parabolic growth model describing the surface reaction step and diffusion step [50]. In the study [51], the reaction between flat Al chips and NaOH solutions was described with the planar one-dimensional shrinking core model (surface chemical reaction step) and the parabolic law (mass transfer rate step). In the case of Al can flat plates and an aqueous solution of sea water, HCl, and Na_2_MoO_4_ (promoter), the reaction was fitted with the model derived from the mentioned improved ‘shrinking core’ model [52]. The dimensional changes in the Al sample reacting with 7.5 M NaOH were illustrated with a contracting volume equation for a parallelepiped [53]. According to the results reported in [54], for composite powders (AZ33 ball milled with C, Co, Bi, Cu, Ni, and SiO_2_), their kinetic curves were fitted with the Avrami–Erofeev equation.

As one could expect, for flat samples, the simulation models were simpler and generally provided a more accurate fitting. The reaction rate constants and diffusion coefficients can be derived from the approximation of experimental results, which can be further implemented for the numerical simulation of the process. As the reaction between Al or Mg and water is exothermal and the reaction rate is temperature-dependent, calculations of the heat power and temperature distribution can contribute a lot to the safe and reliable operation of hydrogen production units fueled with Al- or Mg-based materials and water or aqueous solutions. Promising methods for the preparation of bulk Mg or Al-based composite hydroreactive materials are melting with additives (Cu, Fe, Sn, Ni, etc.) [55,56,57,58,59,60] or ball milling with additives with further powders compacting into pellets [53,61,62]. The known techniques for the effective coalescence of aluminum powder particles (with spherical or irregular shapes) include uniaxial compression [63,64]; cold compacting at 200–600 MPa with further sintering at 500–650 °C [65,66,67,68,69]; spark plasma sintering, commonly under 20–50 MPa and 400–600 °C [70,71,72,73]; and cold pressing at ~600–8000 MPa [67,74]. The reported densities of the compacted samples achieved ~94–99% for hot uniaxial compression, ~92–98% for cold pressing, 98–99% for hold compacting and sintering, and over 99% after spark plasma sintering.

The present study is intended to investigate the hydrogen evolution performance of the pellets (tablets) manufactured from aluminum scrap and copper powder by ball milling with further compacting via cold pressing and spark plasma sintering and reacting with an aqueous salt solution. The parameters of ball milling, scrap and powder compositions and dispersion, and aqueous solution concentration were derived from a previous study [75]. The impact of compacting techniques and temperature on the reaction kinetics will be revealed.

## 2. Materials and Methods

### 2.1. Original Materials and Samples Manufacturing

The major component of the composite hydroreactive samples was scraps of D16 grade aluminum alloy [76], with its elemental composition generally matching that of 2024 aluminum alloy [77,78]. The additive (10 wt.%) was 50–70 μm PMS-1 grade copper powder (99.5%, ‘Rushim.Ru’ Ltd., Moscow, Russia) [79]. And the aqueous solution contained 2 M chemically pure AlCl_3_·6H_2_O (Technical Specification No. 2-191-10, ‘Component-Reaktiv’ Ltd., Moscow, Russia) diluted with distilled water.

The composite powder was manufactured using a centrifugal ball mill (S 100; ‘Retsch’ GmbH, Haan, Germany) and a 125 mL milling pot filled in a glove box (G-BOX-F-290; ‘FUMATECH’ Ltd., Novosibirsk, Russia) under pure argon (99.993%, NII KM’ Ltd., Moscow, Russia) [80]. Ball milling was performed for 1 h using a set (7 pcs.) of 15 mm stainless steel balls with a ball-to-powder ratio of 24:1 and a unidirectional rotation speed of 580 rpm. Large particles (exceeding 1000 μm) were separated from the powder using a sieve shaker (SS 207/B09, ‘Technotest’ S.r.l., Modena, Italy).

Cold pressing of the samples was carried out under a load of 10.0 ± 0.2 tons using a floor-standing hydraulic manual press with a 12-ton capacity (Zubr PGN-12 Professional 43072-12, CJSC ‘Zubr OVK’, Iksha, Russia) and a cylindrical steel mold 10 mm in diameter under a compacting pressure of ~(1.25 ± 0.03) GPa. Spark plasma sintering was carried out in a spark plasma sintering system (Labox 650, ‘Sinter Land Inc.’, Nagaoka, Japan) under the mechanical pressure of 50 MPa in vacuum; three temperatures of 400, 450, and 500 °C were tested (‘pilot’ samples were 15 mm in diameter); for all samples, a heating rate of 100 °C/min. and a holding time of 2 min. were implemented. The tablets for the experiments were sintered at 400 °C (selected temperature) using a graphite die with four 7.8 mm cylindrical channels with pistons. The upper and lower surfaces of the plasma-sintered and cold-pressed tablet-shaped samples were polished with 150-, 600-, and 1000-grit sandpaper to remove traces of graphite paper and surface irregularities. Then, the samples were cleaned with 95% ethanol. The masses of the cold-pressed and plasma-sintered tablets were (0.7845 ± 0.0044) and (0.4233 ± 0.0014) g, respectively. Weighing of the samples was carried out using an analytical balance (VL–220S, ‘NPP Gosmetr’ Ltd., Saint Petersburg, Russia). The final step of tablet preparation was covering them with ultraviolet-curing adhesive acrylic resin (SM Chemie 501) with further solidification under an ultraviolet lamp. The resulting samples had only their top surfaces exposed to ambient media while the rest of the surface was insulated.

### 2.2. Samples Characterization

The samples of aluminum scrap, copper powder, ball-milled composite material, and compacted tablets were investigated via X-ray diffraction (XRD) analysis performed with a ‘Difraey 401’ diffractometer (‘Scientific Instruments’ JSC, Saint Petersburg, Russia) with Cr-Kα radiation (0.22909 nm) with the Bragg–Brentano focusing geometry. The scanning was performed in the 2θ angular range from 14 to 140° (step size 0.01°). The XRD patterns were identified using a database PDF-2 (Powder Diffraction File™) from the International Centre for Diffraction Data (ICDD). The microstructure of the samples was inspected using the scanning electron microscopy (SEM) method in secondary electron (SE) and backscattered electron (BSE) modes. The elemental composition of the samples was evaluated using the energy-dispersive X-ray spectroscopy (EDX) method (operating voltage 20.0 keV). SEM-EDX analysis was performed with a scanning electron microscope (TESCAN VEGA3, ‘Oxford Instruments’ PLC, Abingdon, UK). For the ball-milled composite powder, its particle size distribution was estimated. For the sieve analysis, a vibrating sieve shaker (SS 207/B09, ‘Technotest’ S.r.l., Modena, Italy) with a set of three laboratory test sieves (‘Vibrotechnik’ Ltd., St. Petersburg, Russia) with the standard woven wire mesh opening sizes of 1000, 500, and 100 μm was used. Prior to the analysis, the total mass of the powder sample and masses of the sieves (and bottom pan) were measured using a precision balance (EG 4200-2NM, ‘KERN & SOHN’ GmbH, Balingen, Germany) with a 0.01 g readout. Upon the completion of the ‘sieving’ cycle (shaking for 30 min), the masses of all sieves and the bottom pan were measured again. Then, for the sieves and the bottom pan, the ratio of their ‘surplus’ masses to the total powder sample mass was calculated. And the images illustrating the compacted samples (prior to and during an experiment) were captured with a digital camera (Nikon D5200, ‘Nikon Corporation’, Shanghai, China) with a lens (AF-S DX Micro Nikkor 40 mm f/2.8G, ‘Nikon Corporation’, Shanghai, China).

### 2.3. Test Facility and Experimental Procedure

**Figure 1:**
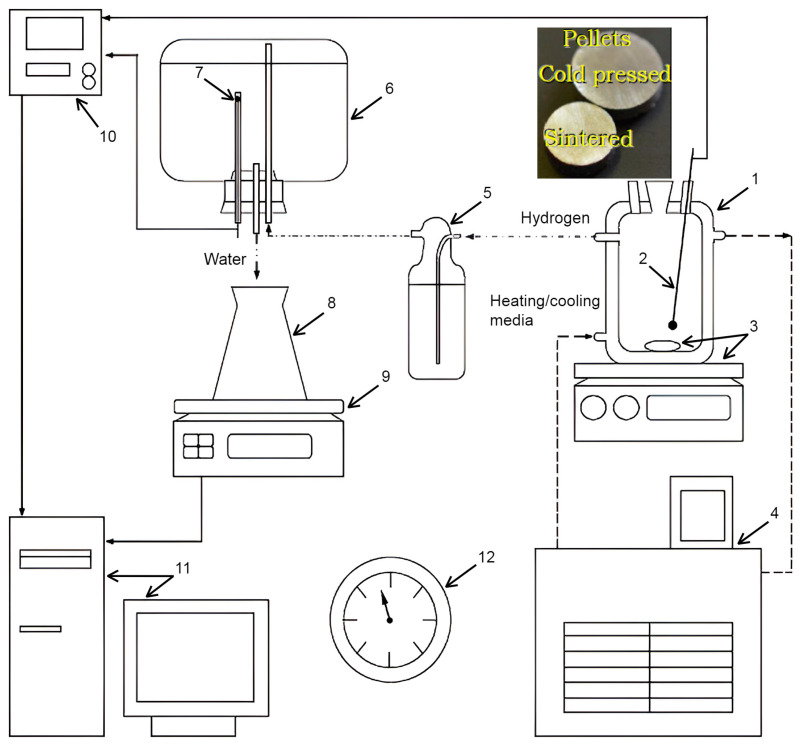
Compacted pellets and experimental set: 1—reactor; 2—thermocouple; 3—magnetic mixer and stirring bar; 4—thermostat; 5—Drexel flask; 6—glass vessel; 7—resistance temperature detector; 8—flask; 9—scales; 10—multichannel thermometer; 11—computer; 12—barometer [13].

The prearrangements for the experiments included filling a reactor (1000 mL, JSC ‘Lenz Laborglas’, Wertheim, Germany) with 1000 mL of the solution and heating it up to 60, 70, or 80 °C with a heater (CC-308B; JSC ‘ONE Peter Huber Kältemaschinenbau’, Offenburg, Germany) under stirring (300 rpm) with a magnetic mixer (C-MAG HS 7; JSC ‘IKA-Werke’, Staufen, Germany). Then the stirring was stopped, and a holder with a sample was mounted in the reactor. Hydrogen released during the reaction passed through a Drexel flask into a glass vessel containing water. Under the incoming gas pressure, water was ejected into a flask. Its mass was continuously measured with scales (ATL-8200d1-I; ‘Acculab Sartorius Group’, New York, NY, USA), and the readings were transmitted to a computer. The temperatures in the reactor and glass vessel were measured, respectively, with an L-type thermocouple (TP.KhK(L)-K11; ‘Relsib’ LLC, Novosibirsk, Russia) installed in a glass protecting tube and a Pt100-type resistance temperature detector (TS-1288 F/11; ‘Elemer’ LLC, Podolsk, Russia) connected to a multichannel thermometer (TM 5103; ‘Elemer’ LLC, Podolsk, Russia). The atmosphere pressure was indicated by a barometer (BTKSN-18; Technical Specification No. 1-099-20-85, ‘UTYOS’ JSC, Ulyanovsk, Russia). The scheme of the test facility and compacted pellets is depicted in Figure 1.

### 2.4. Experimental Data Processing

For each time point, the hydrogen volume at standard conditions (101,325 Pa, 0 °C [81]) from the recorded data sets was derived using the ideal gas law. Hydrogen yield (in %) was obtained by dividing the calculated hydrogen volume values by the hydrogen volume corresponding to the entire oxidation of the hydroreactive material in the sample (see Equation (1)).

(1) $$\alpha \left(\tau \right)=\frac{V(\tau )}{V_{theor}},$$

here τ—time point (s); α—hydrogen yield (% or dimensionless figure from 0 to 1); *V*—calculated volume (L); *V_theor_*—theoretical volume (L).

At each of the tested temperatures, experiments were repeated three times for the samples of both types. Each of the three data sets was used for the calculation of mean values and standard deviations, which were used for plotting kinetic curves with error bars.

Since only one surface of each tablet was exposed to the solution, the resulting kinetic curves were fitted with a one-dimensional contracting geometry model in accordance with Equation (2) [82]:

(2) $$\alpha \left(\tau \right)=k\cdot \tau ,$$

here *k*—reaction rate constant (s^−1^).

From fitting the kinetic curves obtained under different temperatures, the values of *k* were derived. Those values were used for the determination of the activation energy from a graph based on the Arrhenius equation (Equation (3)):

(3) $$\ln k(T)=\ln A-\frac{E_{a}}{R}\cdot \frac{1}{T}$$

where *A*—collision frequency factor (s^−1^); *E_a_*—activation energy (J·mol^–1^); *R*—universal gas constant (8.31 J·mol^–1^·K^–1^); *T*—reaction temperature (K).

The hydrogen volume corresponding to the entire oxidation of the hydroreactive material in the duralumin scrap needed to be obtained to calculate the hydrogen yields. Since the relevant standard stated some tolerated limits for the contents of the alloy’s elements, and since the available EDX method provided us with nothing more than estimations, a scrap sample was tested with a 5 wt.% HCl solution at room temperature, and the resulting hydrogen output was measured. Three tests were conducted in total, providing (1188 ± 6) mL (Standard DIN 1343: 101,325 Pa, 0 °C) per scrap gram on average.

## 3. Results and Discussion

### 3.1. Phase Composition

**Figure 2:**
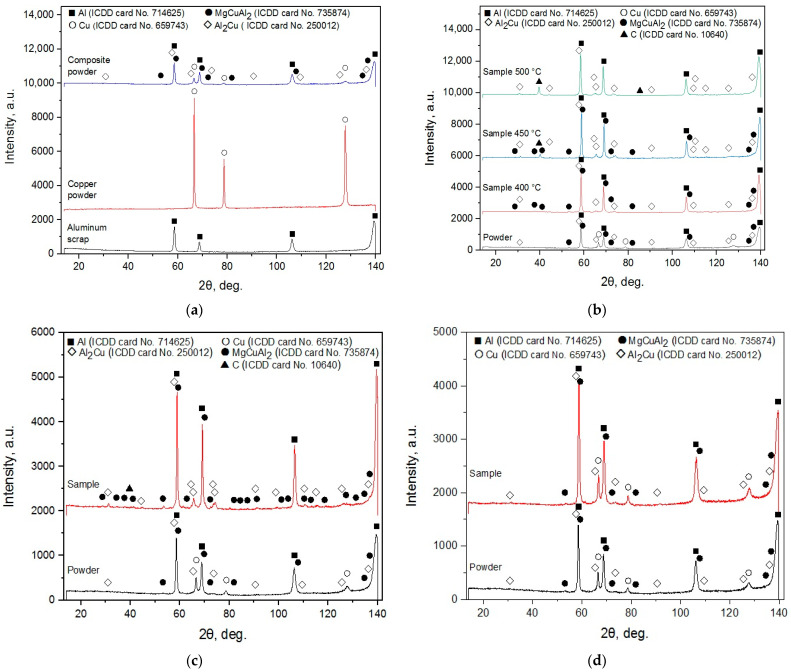
XRD patterns for different samples: (**a**) composite powder; (**b**) samples sintered at different temperatures; (**c**) sintered tablet for experiments; (**d**) cold-pressed tablet for experiments.

The XRD patterns for the ball-milled composite powder, samples sintered at different temperatures, and sintered and cold-pressed pellets for the experiments are shown in Figure 2. For the powder, the major phases were Al and Cu, and the minor were Al_2_Cu and MgCuAl_2_ which emerged from high-energy ball milling. These results diverged from those obtained for the same powder in a previous study [75], wherein Al, Cu, and Al_2_O_3_ were detected. Such a discrepancy could be explained by better purging of the original aluminum scrap, copper powder, and milling balls loaded in the milling pot with Ar, so Al oxidation products fell beyond the detection limits. Although Cu and Mg were essential elements of the D16 aluminum alloy, none of them were detected in the scrap. Natural aging of Al–Cu and Al–Mg–Cu alloys at room temperatures were known to be associated with the emergence of Guinier–Preston (GP) zones enriched with Cu and Guinier–Preston–Bagaryatsky (GPB) zones rich with Cu and Mg atoms which populated {100}_Al_ planes of an Al matrix with the formation of platelets and rods, respectively. At elevated temperatures, those zones turned into the sources for metastable Al_2_Cu and MgCuAl_2_ compounds’ precipitation with further transformation into stable phases. The detection of GP and GDP zones in polycrystalline powder samples was hindered due to the small (a few nanometres) size of the atom clusters and, therefore, non-recognizable or diffuse spots in the XRD patterns [83,84,85,86,87].

For the ‘pilot’ samples sintered at 400 and 450 °C, Al, Al_2_Cu, and MgCuAl_2_ crystalline phases were identified, while holding at 500 °C caused the MgCuAl_2_ to disappear. The peaks of the intermetallic phases were higher than those of the original powder. The samples treated at 450 and 500 °C were found to be contaminated with graphite that came from the graphite die set and graphite paper used for spark plasma sintering. Apparently, the samples that were scanned by the diffractometer lacked intense cleaning. Upon identification of the carbon peaks in the X-ray diffraction patterns, the omission became obvious, and the samples intended for experiments were cleaned more thoroughly by polishing, wiping, and ultrasound treatment in ethanol to remove graphite residuals. No copper was detected for all samples obtained with spark plasma sintering. In the study [74], ball milling of an Al-Cu powder mixture led to the formation of a metastable Al_4_Cu_9_ and stable Al_2_Cu compounds, and the metastable phase was transformed into the stable phase after calcination at 500 °C. Probably, the same explanation could be applied to the MgCuAl_2_ elimination under the same temperature.

The XRD pattern of the sintered tablet for experiments was similar to those for the ‘pilot’ samples manufactured at 400 and 450 °C, although it was smaller in size. No peaks for Cu were observed, while newly formed Al_2_Cu and MgCuAl_2_ phases and contamination with C were distinguished. As to the cold-pressed tablet for experiments, its XRD pattern was very similar to that of the composite powder regarding the correlation between peak heights. The intensities of the peaks for the cold-pressed sample, however, were higher than those for the powder. Such an effect can be attributed to texturization development, which was an example in the study [88], wherein unidirectional pressure applied to Li foil caused its surface to texture.

### 3.2. Microstructure

**Figure 3:**
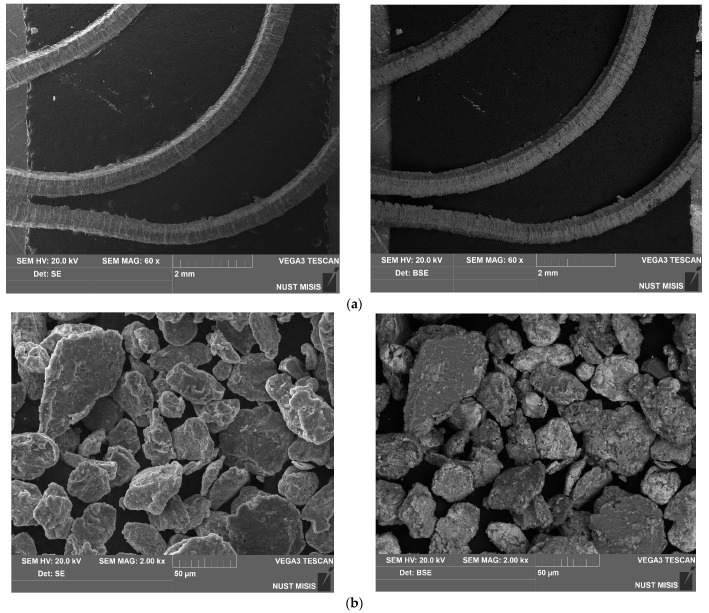

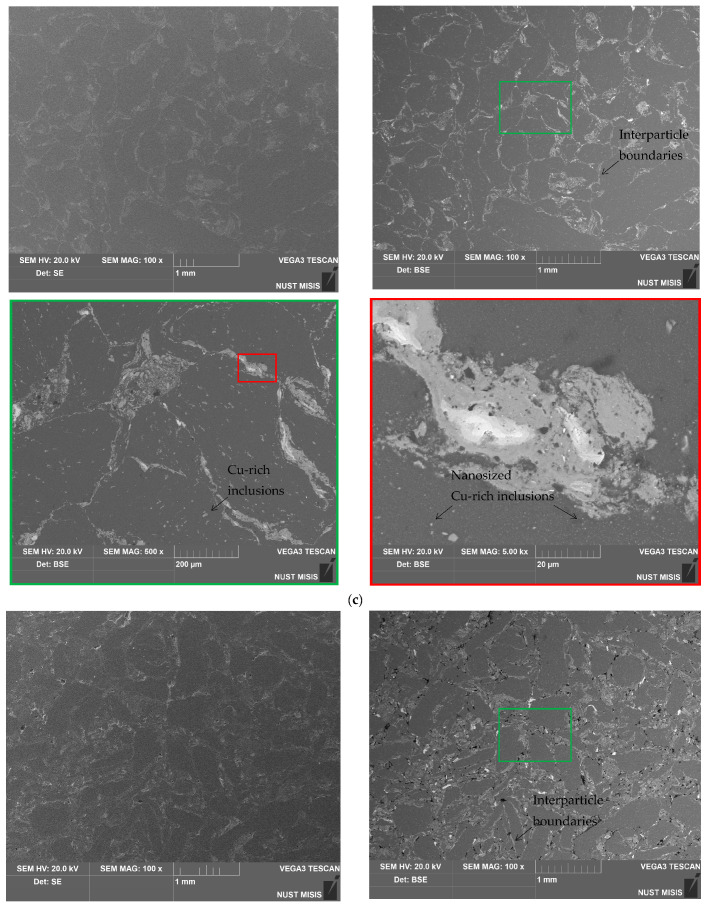

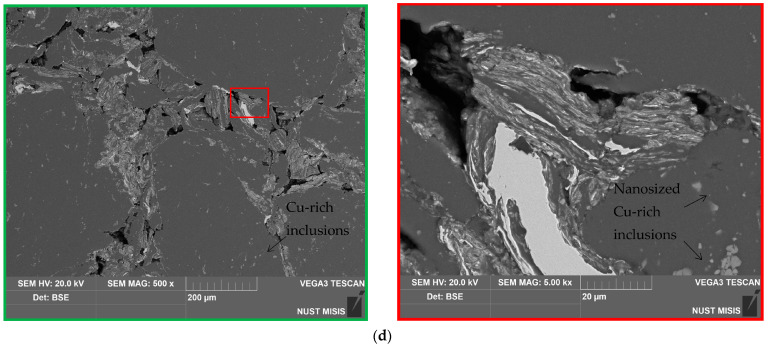
SEM images (SE on the left and BSE on the right) for different samples: (**a**) aluminum scrap; (**b**) composite powder; (**c**) sintered tablet; (**d**) cold-pressed tablet.

The SEM microphotographs of the aluminum scrap, ball-milled composite powder, sintered and cold-pressed tablets captured in the SE and BSE modes are shown in Figure 3. In the images of the scrap particles, traces of mechanical processing (grooves, scratches, rough edges) can be observed. The reshaping of the composite powder particles by the impacts from steel balls included particles fracturing due to local strain hardening and embrittlement, particles flattening, and agglomeration of smaller metal pieces into larger lamellar structures and nearly equiaxed solid shapes. In the BSE powder photograph, the dark grey color corresponds to the base material (D16 aluminum alloy) while light grey and white shades indicate the regions containing heavier metals: sites enriched with copper from the additive and local iron contamination from steel balls.

The images of the sintered tablet reveal that it contained some amount of voids. The structure of the tablet visually reminds of that typical for some alloys with grains of the primary phase (Al, shown in dark grain) and secondary phase (apparently, intermetallic phases Al_2_Cu and MgCuAl_2_, shown in light grey and white) segregated onto the grain boundaries, immensely enlarged, however. The ‘grains’ (scrap particles) had an inhomogeneous nature due to the numerous micro- and nanosized spots of copper-rich phases ‘incrusted’ into them. The cold-pressed tablet was more porous than the sintered one. Again, the structure was composed of dark grey aluminum ‘grains’ with light grey and white ‘grain boundaries’ (interparticle boundaries) and micro- and nanosized ‘inclusions’ enriched with copper. The XRD analysis data demonstrate a relatively small fraction of the newly formed Al_2_Cu and MgCuAl_2_ compounds and observable amount of Cu. Therefore, it could be assumed that, in the case of the cold-pressed pellet, the ‘boundaries’ were majorly formed by the adjacent surfaces of the powder particles, and, due to a relatively short ball milling duration, the ‘surface concentration’ of Cu prevailed over the ‘bulk concentration’. In the case of the sintered tablet, those adjacent Cu-rich surfaces provided copper atoms for the formation of the intermetallides in their vicinity.

The particle size ranges evaluated via the screen analysis are given in Table 1. According to those evaluations, the composite powder mainly constituted of the 100–500 μm particles and those smaller than 100 μm.

### 3.3. Elemental Analysis

**Figure 4:**
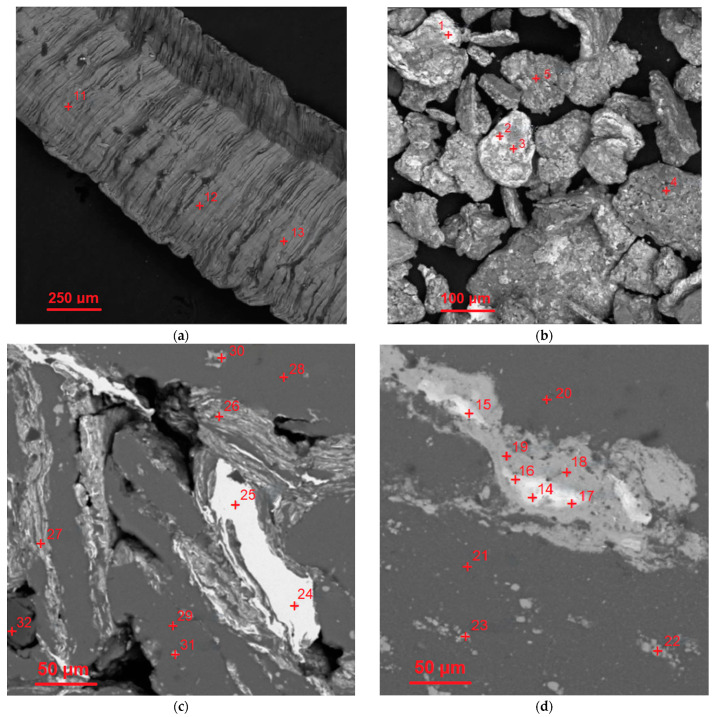
SEM microphotographs (BSE mode) with the marked points scanned using EDX for the starting materials and compacted samples: (**a**) aluminum alloy scrap; (**b**) composite powder; (**c**) cold-pressed pellet; (**d**) sintered pellet.

**Figure A1:**
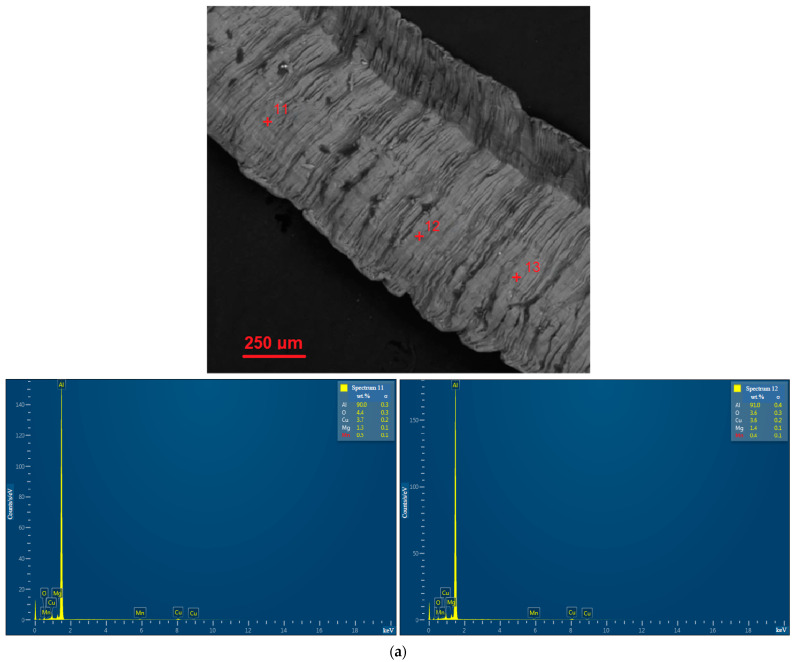

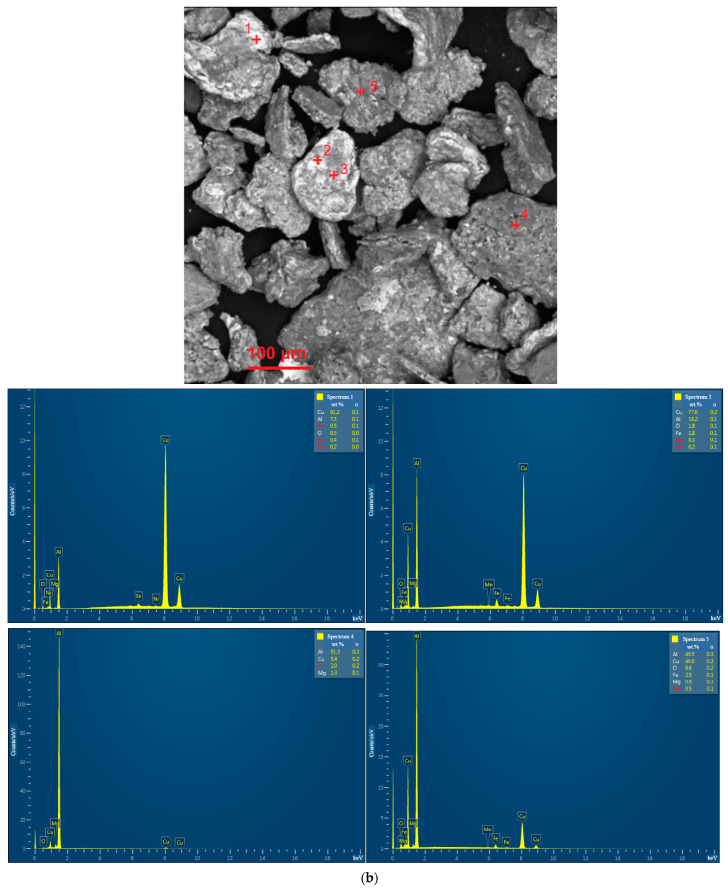

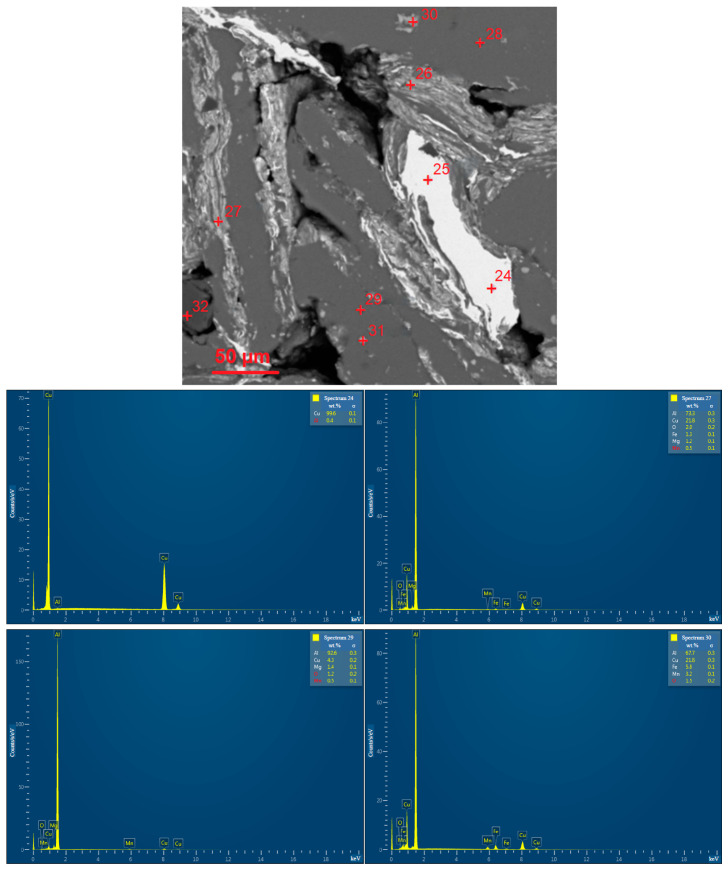

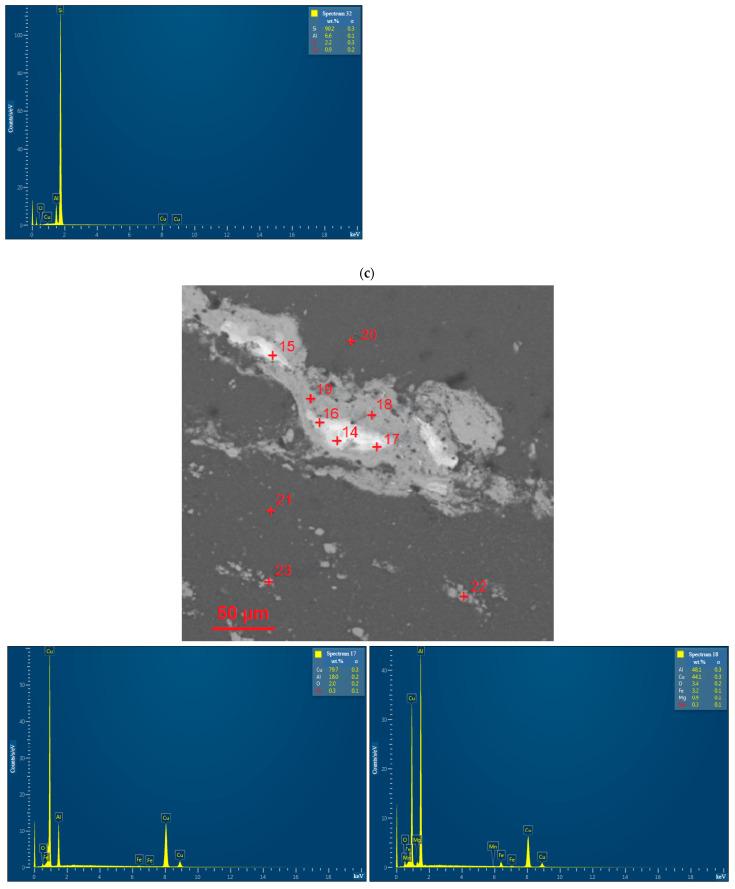

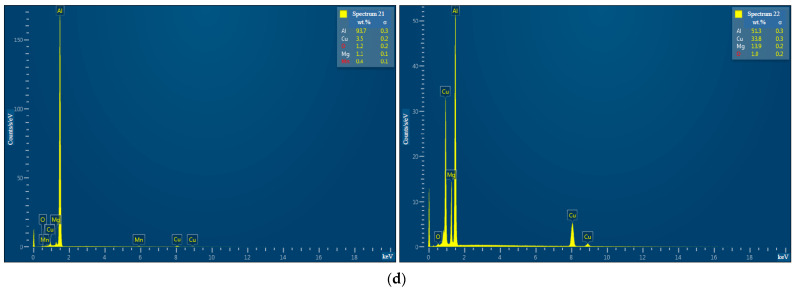
EDX analysis results (spectra) at the selected points for the starting materials and compacted samples: (**a**) aluminum alloy scrap; (**b**) composite powder; (**c**) cold pressed pellet; (**d**) sintered pellet.

The results of the EDX analysis for the scrap sample, composite powder, and cold-pressed and sintered tablets at the selected most representative points are summarized in Table 2. The respective scanned points are depicted in the BSE microphotographs in Figure 4 (wherein the entire sets of investigated points are marked). The EDX spectra for those selected scanned points are arranged in Figure A1a (see Appendix A). According to the represented data, the scrap was composed of Al, Cu, Mg, and Mn—elements typical for the 2024 aluminum alloy grade. The composite powder contained detectable amounts of Fe and Ni. Although these elements could be found as impurities in the D16 aluminum alloy, their higher quantities are likely associated with the contamination from the milling balls (AISI 304 steel alloy). For the cold-pressed tablet, the basic alloy components (Al, Cu, Mg, and Mn) and Fe and Si (typical elements of both D16 and AISI 304 alloys) were observed. The same set of elements was found in the sintered tablet. All the samples contained some amount of O as well.

From the examination of all of the obtained EDX patterns and their matching regions on the SEM images (in BSE mode), it was established that the white areas within the interparticle boundaries for both sintered and cold-pressed pellets were highly enriched with Cu (70.4–99.6 wt.%). The dark grey regions contained over 90 wt.% of Al. Some of the light grey areas (both inter- and intraparticle spots) were composed of (21.8–44.1) wt.% Cu and (48.1–73.3) wt.% Al, and at one of the tested points, 13.9 wt.% of Mg was detected as well, while the other areas (intraparticle spots) of the same shade contained only up to (6.3–7.6) wt.% Cu. It could be assumed that some of the intraparticle ‘inclusions’ observed on the microphotographs were generally composed of the intermetallic Al_2_Cu and MgCuAl_2_ phases while the other ones represented the sites enriched with Cu.

### 3.4. Reaction Kinetics

**Figure 5:**
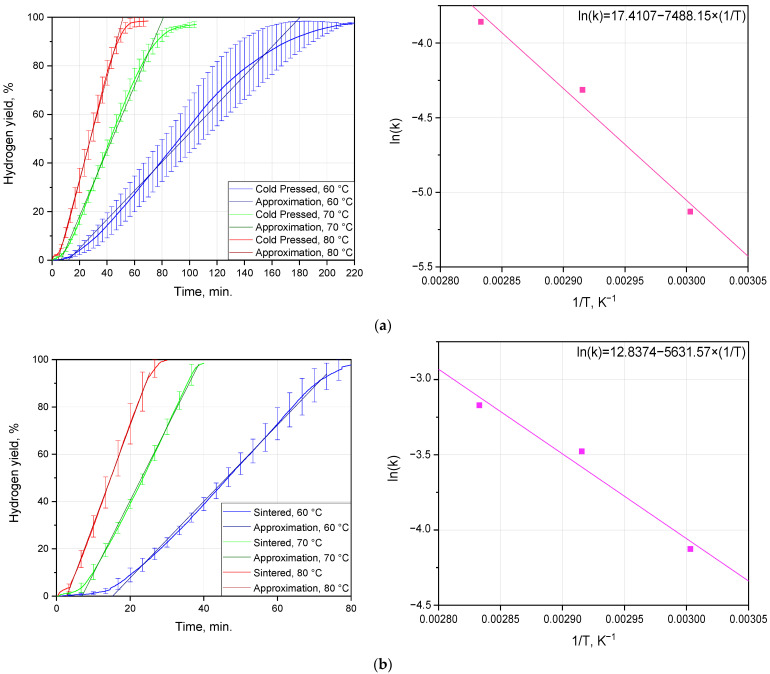
Hydrogen evolution kinetic curves fitted with linear functions and Arrhenius plots: (**a**) cold-pressed tablets; (**b**) sintered tablets.

The experimental results on the hydrogen evolution kinetics at different temperatures for the cold-pressed and sintered tablets are depicted in Figure 5 and listed in Table 3. As can be seen from the plots, all kinetic curves had an ‘S’-shape typical for topochemical reactions, with an acceleration part at the beginning, a deceleration portion at the final stage, and a large section of a nearly constant reaction rate. Due to the extended sections of the nearly constant reaction rate, the contributions of the initial and final curve portions relating correspondingly to nucleation spreading over the surface exposed to the solution and the non-uniform shrinking of the reacting surface area of the residual sample portion were neglected. According to the remarks from [82], the starting gas evolution (1–3%) can be excluded from the analysis of the said kinetic curves. In the present study, for a more accurate approximation of the kinetic curves, portions of 3–93% hydrogen yield were selected. As was in more detail discussed in a previous study [75], the reaction between Al-based samples and the AlCl_3_ aqueous solution with hydrogen generation resulted in the formation of a hydroxychloride compound, Al_m_(OH)_n_Cl_3m−n_ (m ≥ 1; 0 < n ≤ 3 m), highly soluble in water. As no solid reaction product originated during the process, diffusion effects fell out of consideration, and the one-dimensional contracting geometry model was used for fitting the experimental data sets.

According to the plots, the eventual hydrogen yields for both (cold-pressed and sintered) sorts of tablets obtained under all of the tested temperatures were close to 100%. Deviations from that value (as well as statistical errors) could arise from the potential non-uniformity in the distribution of the Cu additive over the ball-milled powder particles and then over tablets, the presumptive existence of the abovementioned GP and GPB zones enriched with Cu and Mg, and the presence of oxidized aluminum in the samples. The resulting hydrogen output per mass unit definitely comprised contributions from the reacted Al and Mg and, probably, some contributions from minor impurities. Partial oxidation of the samples was proven by the EDX data. However, since the standard arrangement of the EDX equipment and procedure does not provide a direct measurement of the light element content (the samples could potentially be contaminated with O and, to a lesser extent, with C) [89,90], we had no precise, reliable data on the chemical composition of our samples. Therefore, any unaccounted contributions of contaminating elements could reduce the actual mass of Al (and, to a much lesser extent, Mg) in the samples, and their final hydrogen yields obtained in the experiments could fall below the expected outputs per mass unit of the scrap in the samples. If, due to any non-uniformities in the distributions of the alloying elements and impurities of the scrap, our ‘acidic estimation’ of hydrogen output was underestimated, it also could contribute to the deviations in hydrogen yields, making them higher. Thus, discrepancies in the actual compositions of the pellets could affect the estimation accuracy for the aluminum contents in the samples.

Due to the different sizes of the cold-pressed and sintered tablets, a comparison between their hydrogen generation performances was barely possible. But it was still noteworthy that the sintered pellets reacted considerably faster than the cold-pressed ones. Thus, the kinetic curves for the sintered tablets reacting at 60, 70, and 80 °C achieved the plateau in nearly 80, 40, and 30 min., respectively, while the corresponding time intervals for the cold-pressed pellets were approximately 220, 100, and 70 min. Another mentionable observation related to the fitting accuracy, which was definitely higher in the case of the sintered samples. That effect could be associated with a variety of factors. One of them could be the distinguishable porosity of the cold-pressed sample (depicted by the SEM images), which resulted in changing the actual reaction surface area during the experiments. Another one could be caused by the bigger size of the cold-pressed samples and the larger deviations of the reaction temperature from the specified isotherms due to heat emission from the reaction (the temperatures of the solution in the reactor measured during experiments confirmed a larger maximum increase for the larger samples, of ~0.4 °C, over that for the smaller ones, of ~0.2 °C).

The reaction rate constants were derived for each tested temperature from the approximation of the kinetic curves with linear functions. Those values were used for the graphical determination of the activation energy values in accordance with the Arrhenius equation. The calculated activation energies for the cold-pressed and sintered tablets were (62.23 ± 9.11) and (46.80 ± 8.87) kJ/mol.

**Figure 6:**
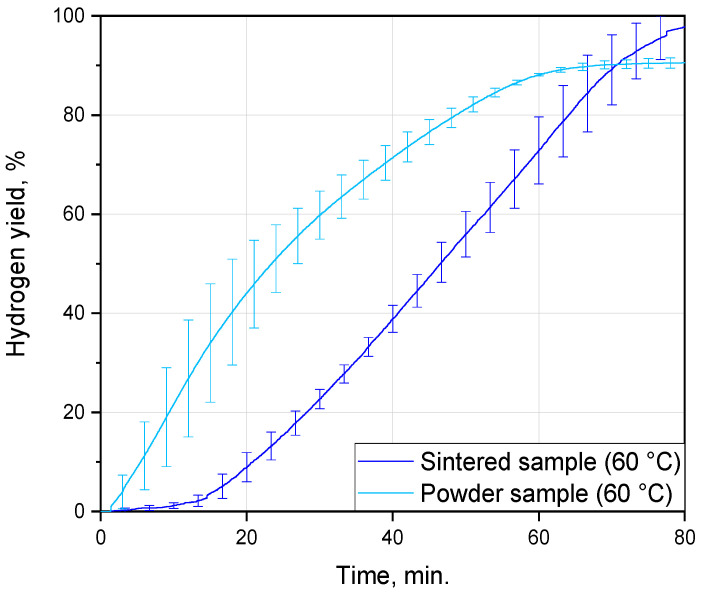
Comparison of the hydrogen evolution kinetic curves for the cold-pressed tablets and relative powder from a previous work [75].

From the comparison of the kinetic curves obtained for the sintered samples in the present study and for the composite powder from the preceding study [75], it can be seen that the powder reacted faster, as expected (see Figure 6). Both samples were manufactured from the same D16 alloy scrap and 10 wt.% copper powder of the same grade, ‘POS-1’, under the same ball milling parameters and tested in the same solution at the same temperature of 60 °C. For both the sintered tablets and composite powder, the reaction was completed after nearly 70–80 min. The samples had different phase compositions (Cu and Al_2_O_3_ in the powder vs. MgCuAl_2_ and CuAl_2_ in the tablet), whose influence on the local galvanic corrosion of aluminum might vary depending on their standard electrode potentials (under the tested conditions). The reaction conditions were not the same—stirring in the case of the powder and its absence for the tablet, and a drastic difference in the sizes of a particle and a compact (partially sealed with compound)—which could affect heat transfer from the surfaces of the reacting samples into the solution and, therefore, the temperature profile within the interface between them. The powder and tablet samples could differ in the amounts of strain energy accumulated during their processing, which might also impact the severity of aluminum corrosion with hydrogen evolution. The crystalline grain size could affect corrosion as well, although it was not yet clearly established whether fine or coarse grains were more vulnerable to corrosion attacks [91]. Those characteristics could be evaluated from their XRD patterns, provided the tablet sample was ground into powder and a diffractometer with a higher resolution was used.

**Figure 7:**
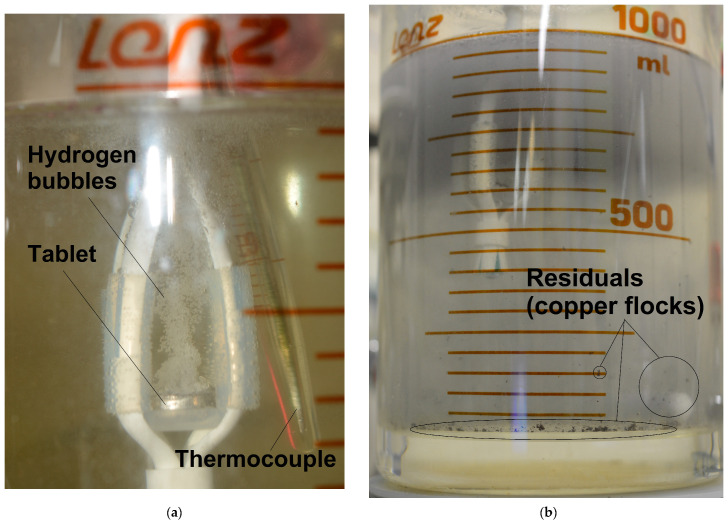
Illustrations during the reaction process and after it: (**a**) reacting cold-pressed tablet; (**b**) solid residuals (copper flocks).

In the present study, the tablet samples were scanned in the form of polished bulk samples. The penetration depth of the Cr-Kα radiation for Al was nearly ~20 μm [92], while the thickness of the texturized tablet portions (from top to bottom) was not established in the present study. If it exceeded that of 20 μm, the evolution of the basic aluminum XRD peaks from the powder to the compacted pellets was likely a result of texturization. From time to time during the reaction, pieces of copper (in the form of flocks) were ‘ripped out’ of the sample by the two-phase flow of hydrogen bubbles and solution. Those brown copper-based flocks were solid residuals remaining upon completing the reaction (see Figure 7b). The residuals remaining after testing the same powder, analyzed in a previous study [75], were composed mainly of Cu with some amounts of Cu_2_O and Al_2_O_3_ phases. Although the reacting tablets (both cold-pressed and sintered) vigorously evolved hydrogen bubbles that floated upwards, entraining the solution and forming massive, seemingly turbulent flows which could provide effective heat removal by intensive mixing (see Figure 7a), that version is to be proved by another investigation focused on heat and mass transfer problems.

In the study [93], the spark plasma sintering method was used for the consolidation of aluminum scrap (AA6061 grade with Al, Mg, and Cu) chips into billets (processing at 200 MPa, 490 °C). The examination of the samples revealed that the sample had a lamellar structure; Al chips were majorly welded to each other as alumina layers onto their surfaces got disrupted; some amount of micro-pores (5–20 μm) and entrapped air were detected; and all areas depicted in light shades (elongated regions along grain boundaries and separate spots) were enriched with copper and formed intermetallic compounds. It was supposed that SPS treatment provided partial fracturing of the surficial Al oxides, entrapped gas adsorption, and activation of the metallic surface. According to the results reported in [94], compacted samples obtained from the 2024 alloy (80, 50, 20, and 5 μm powders) using the SPS method (50 MPa, 500 °C) immersed in a 3.5 wt.% NaCl aqueous solution experienced severe corrosion attacks in the vicinity of the intermetallic phase (Al_2_Cu and MgCuAl_2_) inclusions. Those phases were mostly located at the grain boundaries, but they were observed inside the grains as well in the form of well-distributed needle-shaped or round spots dozens of nanometers in size and decentralized speckles along the sub-grain boundaries. The predominating processes were disintegration of the passivation aluminum oxide film, galvanic corrosion of aluminum leading to the evolution of trenching around the Cu-rich phase (the potential of which was higher than that of the matrix), re-passivation due to the accumulation of the reaction products, disruption of the re-passivation film, formation of separate pits, and their merging. Thus, the ‘successful performance’ of the sintered tablets could be ascribed to the disintegration of the passivation films onto the powder particles via heat treatment in a vacuum under pressure and the formation of copper-rich sites favoring anodic dissolution of the adjacent aluminum regions in an aggressive chlorine solution with hydrogen evolution.

Although it was likely that spark plasma sintering provided superior results over cold pressing, it would still be interesting to improve the compacting output of the latter technique by combining it with moderate heating up to 100–300 °C. Due to increased aluminum formability at elevated temperatures, such warm compacting techniques provided a higher density of the compacted samples [95,96,97]. It would be interesting to compare hydrogen generation performances (yield and evolution rate) for equal-sized samples manufactured using spark plasma sintering and warm compacting. Also, it could be useful to manufacture compacted and sintered samples without additives and a sample with the same amount of Cu additive produced using conventional melting for further testing and comparison so that the influence of the accumulated strain energy, crystal phase composition, and microstructure could be clarified. According to the findings from [98], larger cathodic sites (Ni) were beneficial for Mg corrosion with hydrogen evolution in KCl solutions, while the results from [99] demonstrated the superiority of nanosized Bi cathodic spots over the micron-sized ones for Al powders reacting with tap water. Taking that into account, the effect of the ‘cathodic’ site size is another characteristic that could be of interest.

Despite the fact that powder samples react faster, their ‘compacted alternatives’ possess a number of advantages. If the reaction in a hydrogen supply unit (imaginable) is growing uncontrollably, they can be retrieved from the reaction zone, or the solution can be drained or pushed out into a fluidly coupled volume, thus reducing their contacting area. Moreover, provided that an approximately isothermal mode is maintained, specific (per surface unit) reaction constant rates for the standard bulk shapes (plates, cylinders, etc.) could be determined with good precision, thus contributing to the ‘predictability’ of the reaction process.

### 3.5. Potential for Practical Application: Challenges, and Possible Remedies

The recovery of the additives and alloying elements is a reasonable question (especially if their amounts exceed values typical for small-scale laboratory tests). Mg definitely reacted with AlCl_3_, resulting in the formation of complex compounds. In a preceding study on the low-temperature hydrogen production from Mg and aqueous saline solutions, after the experiment at 0 °C with an excess of Mg, Mg_3_(OH)_5_Cl·4H_2_O and Al_9_(OH)_21_Cl_6_·22H_2_O compounds were identified in the vaporized solution sample [100]. An approach to Mg recovery is to be elaborated. As to Al_m_(OH)_n_Cl_3m-n_ (m ≥ 1; 0 < n ≤ 3 m), its highest basicity of ~83% corresponds to Al_2_(OH)_5_Cl·2H_2_O (Lesukite). It is used for water treatment and the binding of antibacterial agents to cotton fabric [101,102,103]. Under thermal treatment at 543 K, Al_2_(OH)_5_Cl decomposes into Al(OH)_2_Cl, AlOOH, and H_2_O; and at 723 K, Al(OH)_2_Cl decomposes into Al_2_O_3_, HCl, and H_2_O [104]. Thus, aluminum oxidized with an AlCl_3_ solution has the potential to be recovered via the decomposition of Al_2_(OH)_5_Cl and electrolysis of Al_2_O_3_.

The evolution of other, minor, elements is unclear. An individual study is needed with much larger amounts of scrap to trace those elements. If they are not consumed during experiments, they could be separated from the solution by filtering and treated in an ultrasound bath for particle ‘deglomeration’ and intense washing with distilled water. After that, an attempt could be made to separate Fe with a magnet. Si could be removed with a heavy liquid for gravitational separation, for example, sodium polytungstate (density up to 3.15 g/cm^3^), or more commercially available bromoform (maximum density 3.42 g/cm^3^), with caution [105]. For the extraction of Cu and Mn, in the study [106], their dissolution in 2 M H_2_SO_4_ with 10 wt.% H_2_O_2_ was proposed, with further sedimentation of Mn and Cu, correspondingly, with (NH_4_)_2_S_2_O_8_ and organic extractor LIX 84 IC.

Regardless of the fact that no special tests on hydrogen contamination with chlorine were conducted in the present study, the potential for such a ‘bottleneck’ is high. Huge amounts of waste hydrogen delivered by oil refineries, chlor-alkali processes, the chlorate industry, and coal gasification worldwide attract close attention as a potential energy resource. Its large-scale utilization enhances the overall efficiency of other energy systems, for which stationary fuel cell systems are considered a feasible solution [107,108]. The most common contaminants in low-grade hydrogen are N_2_, Cl_2_, O_2_, CO, CO_2_, and CH_4_. Contributions from H_2_S and NH₃ have also been reported. Proton exchange membrane (PEM) fuel cells were proven to recover after short-term contamination with sodium carbonate [109], while contamination with chlorine had the potential to induce severe degradation of Pt catalysts. In studies [110,111], the dynamics of PEM fuel cell performance under the effect of introduced HCl contamination were studied. Various combinations of current densities, operation temperatures, contaminant contents, and relative humidities were tested. The reported results confirmed the deteriorating effects of chlorine entrained by hydrogen and air flows on the catalyst performance at the cathode and anode sides, respectively. The gravity of the contamination impact was independent of the injection side and increased with both current density and HCl concentration. The presence of Cl^−^ caused an increase in the charge transfer and mass transfer resistances on the cathode side. The decrease in the electrochemical catalyst surface area for the cathode was more significant than that for the anode site. The key catalyst poisoning mechanisms were the site-blocking effect of adsorbed Cl^−^ on the Pt surface and the enhancement of its dissolution due to the formation of chloride complexes. A decrease in the relative humidity contributed to the catalyst’s degradation, while lowering the temperature resulted in a delayed fuel cell voltage drop. At the impurity concentration of 0.1 ppm, the cell performance exhibited a steady decay, while its increase to 1 ppm and higher values resulted in an abrupt voltage drop. In the study [112], high chlorine contaminations of 100 and 200 ppm were tested, and at 0.6 V, 94 and 82% losses in performance were established for the anode and cathode. After that, cell recovery was tested by operating the cell at 0.1 V. It was claimed that full recovery of the contaminated cell was achieved and that the anode recovered faster than the cathode due to the replacement of the adsorbed chloride species with positive proton ions.

Although 50 ppm of a chloride salt (AlCl_3_, FeCl_3_, CrCl_3_, NiCl_2_, and MgCl_2_) introduced in the air stream caused much slower cell voltage decay than the same amount of HCl at 80 °C [113], special efforts are to be made to prevent chlorine contamination. Preliminary recommendations could be the maximum possible reduction of the operational temperature and chlorine concentration in the solution, the mounting of an effective condenser at the gas outlet to retain solution moisture entrained by the hydrogen stream, and the analysis of dry hydrogen for chlorine content via a suitable technique. For example, for the determination of the chloride concentration in gas, a spectrophotometric method in accordance with a procedure outlined in [114] could be employed. That procedure includes the bubbling of contaminated hydrogen through methyl orange-based absorbing solution and detecting the change in its color.

## 4. Conclusions

The present study was focused on the testing of tablets manufactured by the ball milling of D16 aluminum alloy scrap with a copper additive and further compacting of the resulting composite powder via cold pressing or spark plasma sintering. From the summarization of the results, the following findings were derived:

- In addition to Al and Cu, the composite powder contained minor amounts of new phases, CuAl_2_ and MgCuAl_2_, and the same set of compounds was detected in the case of the cold-pressed samples, while for the sintered ones, no Cu was identified;
- The microstructure of the sintered and cold-pressed samples was represented by particles of the original scrap with ‘interparticle’ boundaries and micro- and nanosized ‘intraparticle’ inclusions, which were identified as intermetallic CuAl_2_ and MgCuAl_2_ compounds and Cu-rich sites. The zones enriched with Cu promoted galvanic corrosion of the Al matrix in the tested chlorine aqueous solution and thus intensified hydrogen generation;
- Despite the relatively small surface areas of the tablets exposed to the solution and carrying the reaction (as compared to that of the composite powder), their hydrogen yields and evolution rates happened to be quite impressive;
- The kinetic curves for the sintered pellets were approximated with higher precision than those for the cold-pressed ones. The possible reasons for that are a larger size of the latter that resulted in a higher temperature deviation from the isotherms and a large number of substantial voids, which could cause variations in their specific surface area during the reaction.

## Figures and Tables

**Table 1:** Particle size distribution.

Size, μm	Mass, g	Fraction, %
>1000	0.20	0.4
500–1000	2.44	5.0
100–500	37.07	76.5
<100	8.75	18.1

**Table 2:** Elemental compositions for the selected points of the original and resulting samples.

Sample	Spectra No.	Cu	Al	O	Mg	Fe	Mn	Si	Ni
Powder	1	91.2 ± 0.1	7. 3 ± 0.1	0.5	0.2	0.5 ± 0.1	-	-	0.4 ± 0.1
	3	77.6 ± 0.2	18.2 ± 0.1	1.8 ± 0.1	0.3 ± 0.1	1.8 ± 0.1	0.2 ± 0.1	-	-
	4	5.4 ± 0.2	91.3 ± 0.3	2.0 ± 0.2	1.3 ± 0.1	-	-	-	-
	5	40.0 ± 0.3	49.5 ±0.3	6.6 ± 0.2	0.9 ± 0.1	2.5 ± 0.1	0.5 ± 0.1	-	-
Scrap	11	3.7 0.2	90.0 ± 0.3	4.4 ± 0.3	1.3 ± 0.1	-	0.5 ± 0.1	-	-
	12	3.6 ± 0.2	91.0 ± 0.4	3.6 ± 0.3	1.4 ± 0.1	0.4 ± 0.1	-	-	-
Cold-pressed	24	99.6 ± 0.1	0.4 ± 0.1	-	-	-	-	-	-
	27	21.8 ± 0.3	73.3 ± 0.3	2.0 ± 0.2	1.2 ± 0.1	1.3 ± 0.1	0.5 ± 0.1	-	-
	29	4.3 ± 0.2	92.6 ± 0.3	1.2 ± 0.2	1.4 ± 0.1	-	0.5 ± 0.1	-	-
	30	21.8 ± 0.3	67.7 ± 0.3	1.5 ± 0.2	-	5.8 ± 0.1	3.2 ± 0.1	-	-
	32	0.9 ± 0.2	6.6 ± 0.1	2.2± 0.3	-	-	-	90.2 ± 0.3	-
Sintered	17	79.7 ± 0.3	18.0 ± 0.2	2.0 ± 0.2	-	0.3 ± 0.1	-	-	-
	18	44.1 ± 0.3	48.1 ± 0.3	3.4 ± 0.2	0.9 ± 0.1	3.2 ± 0.1	0.3 ± 0.1	-	-
	21	3.5 ± 0.2	93.7 ± 0.3	1.2 ± 0.2	1.1 ± 0.1	-	-	-	0.4 ± 0.1
	22	33.8 ± 0.3	51.3 ± 0.3	1.0 ± 0.2	13.3 ± 0.2	-	-	-	-

**Table 3:** Hydrogen yield, reaction constants, coefficients of determination, collision frequency factors, and activation energies for different experiments.

Sample	Temperature, °C	Hydrogen Yield *, %	Reaction Rate Constant, s^−1^	R^2^	Collision Frequency Factor, s^−1^	Activation Energy, kJ/mol
Cold-pressed Tablet	60	97.66 ± 0.70	5.93 × 10^−3^	0.9885	36.42 × 10^6^	62.23 ± 9.11
	70	97.00 ± 1.27	13.39 × 10^−3^	0.9937		
	80	98.45 ± 2.14	21.12 × 10^−3^	0.9982		
Sintered Tablet	60	97.79 ± 3.62	16.16 × 10^−3^	0.9981	37.60 × 10^4^	46.80 ± 8.87
	70	98.50 ± 2.60	30.92 × 10^−3^	0.9978		
	80	99.71 ± 0.27	42.00 × 10^−3^	0.9991		

* Maximum 100%.

## Data Availability

Data are contained within the article (Buryakovskaya, O.A.; Suleimanov, M.Z.; Vlaskin, M.S.; Kumar, V.; Ambaryan, G.N. Aluminum Scrap to Hydrogen: Complex Effects of Oxidation Medium, Ball Milling Parameters, and Copper Additive Dispersity. *Metals* **2023**, *13*, 185. https://doi.org/10.3390/met13020185 [75]).

## Appendix A

EDX analysis results for the D16 aluminum alloy scrap, ball-milled powder, and cold-pressed and sintered pellets are shown in Figure A1. In the SEM images, all inspected points were marked, while the attached EDX spectra were limited by the selected, most representative spots since many of them had quite similar elemental compositions.
